# Intersections of racism, disability, and postpartum depression in minoritized racial and ethnic populations

**DOI:** 10.1007/s00737-025-01599-6

**Published:** 2025-06-13

**Authors:** Maria McDonald, Genevieve Lyons, Rosemary B. Hughes, Kathryn Laughon, Casey Johnson, Jeanne L. Alhusen

**Affiliations:** 1 School of Nursing, University of Virginia, 202 Jeanette Lancaster Way, Charlottesville, VA, Virginia 22903 USA; 2 Department of Public Health Sciences, University of Virginia, Charlottesville, VA USA; 3 University of Montana Rural Institute for Inclusive Communities, Missoula, MT USA

**Keywords:** Postpartum depression, Intersectionality, Disability, Emotional distress, Racism

## Abstract

**Purpose:**

Using an intersectional lens, this study examined the association between maternal reports of emotional distress due to racism (EDR) and postpartum depression (PPD) symptoms among persons with and without disabilities in minoritized racial and ethnic populations.

**Methods:**

An analysis of Phase 8 (2018–2020) data from the Pregnancy Risk Assessment Monitoring System (PRAMS) included 2,452 respondents with a recent live birth from three states (MO, GA, VA). We examined the association between EDR and PPD symptoms among respondents with and without disabilities using Pearson’s chi-square test. Multivariate regression models were used to further estimate the odds of PPD symptoms associated with EDR and to examine differences in this relationship by disability status.

**Results:**

EDR was significantly associated with PPD symptoms, with a higher prevalence of PPD symptoms among those with at least one disability (52.3%) compared to those without disabilities (19.9%). After adjusting for maternal demographics, EDR was significantly associated with PPD symptoms across disability groups. No significant interaction between disability group and EDR was observed, suggesting the association’s strength did not differ significantly by disability group in this study. In the fully adjusted model, history of depression attenuated the EDR-PPD association across disability groups, highlighting the effect of prior depressive episodes on PPD outcomes.

**Conclusion:**

EDR is significantly associated with PPD symptoms, and persons with disabilities in minoritized racial and ethnic populations may be particularly susceptible to EDR and PPD. Additional research using an intersectional perspective is needed to elucidate PPD disparities as related to multiple, intersecting social identities and experiences of discrimination.

## Highlights

Postpartum depression (PPD) is a common and complex complication of childbirth associated with adverse maternal and child health outcomes.

Disparities in PPD by race, ethnicity and disability status are notable and exacerbated by exposures to racism and systemic discrimination.

Persons with disabilities in minoritized racial and ethnic groups can have an elevated risk of PPD due to discrimination in multiple forms.

More studies using an intersectional lens are needed to understand disparities in PPD among persons with multiple marginalized identities.

Postpartum depression (PPD) is a prevalent and multifaceted complication of pregnancy and childbirth, with important implications for adverse maternal and child health outcomes (Slomian et al. 2019). Clinically characterized by the onset of a major depressive episode within the first four weeks following delivery, PPD affects approximately 13% of postpartum persons in the United States (American Psychiatric Association 2013; Bauman 2020).

Numerous studies have documented the adverse consequences of maternal PPD, which significantly impact parents, infants and families (Slomian et al. 2019). Untreated PPD is associated with a range of maternal consequences, including persistent depressive symptoms, diminished quality of life, impaired maternal-infant bonding, challenges in intimate and interpersonal relationships, increased risk-taking behaviors (such as smoking and substance abuse), and elevated risk of maternal suicidality (Allen et al. 2009; Darcy et al. 2011; Lilja et al. 2012; Paris et al. 2009). Furthermore, untreated maternal PPD can have untoward effects on infants and children, potentially resulting in delays in cognitive, social and emotional development (Feldman et al. 2009; Lubotzky-Gete et al. 2021).

Emerging evidence suggests that persons from historically and currently marginalized racial populations are disproportionately affected by PPD and experience a higher prevalence of PPD symptoms compared to non-Hispanic White persons (Bauman 2020; Getahun et al. 2023). A nationally representative sample of respondents who experienced a live birth in 2018 demonstrated elevated PPD symptom prevalence among non-Hispanic Black (18.2%), non-Hispanic American Indian and Alaska Native (22.0%), and non-Hispanic Asian (19.2%) respondents compared to non-Hispanic White respondents (11.4%) (Bauman 2020). Importantly, social determinants of health, including exposure to racism, limited access to resources, and residing in disadvantaged neighborhoods have been shown to exert a significant impact on maternal mental health outcomes and contribute to disparities in PPD symptoms by race and ethnicity (Bossick et al. 2022; Onyewuenyi et al. 2023).

Approximately 12% of U.S. women of childbearing age have a disability (Courtney-Long et al. education and economic opportunities) which pose important risks for adverse health conditions (Courtney-Long et al. 2017). Disability status is associated with a range of adverse maternal and neonatal outcomes, including increased risk of infection, preterm labor, and low birth weight (Deierlein et al. 2021). Additionally, persons with disabilities are at least twice as likely to experience PPD symptoms as those without disabilities (Alhusen et al. 2023). Importantly, minoritized racial and ethnic populations experience a disproportionately higher prevalence of disability compared to non-Hispanic White persons (Nuru-Jeter et al. 2011; Varadaraj et al. 2021). The combination of disability and membership in a minoritized racial and ethnic groups is associated with especially heightened risk for social and structural disparities (e.g., reduced access to quality healthcare, safe living environments, adequate insurance coverage, education and economic opportunities) which pose important risks for adverse health conditions (Courtney-Long et al. 2017; Horner-Johnson 2020; Tarasoff 2017). Notably, disabled persons from minoritized racial and ethnic groups often experience more racism and discrimination than their non-disabled peers and report worse physical and mental health outcomes (Dorsey Holliman et al. 2023; Mereish 2012).

The theory of intersectionality, first articulated by American legal scholar Kimberlé Williams Crenshaw in 1989, provides an important framework for examining how various forms of oppression, including racism, ableism, sexism, and classism, shape health outcomes for those situated in multiple marginalized groups (Crenshaw 1991). A core tenet of intersectionality theory posits that persons with multiple, marginalized social identities (in this case, minoritized race/ethnicity and disability status) may face unique risks for poor outcomes, particularly in the context of discrimination exposure. While previous studies have examined PPD disparities by race, ethnicity and disability status independently, there is limited understanding of how these intersecting social identities, along with experiences of discrimination, may influence PPD risk. Using the intersectionality framework, this study aims to examine the relationship between exposure to racism and PPD symptoms among persons with and without disabilities in minoritized racial and ethnic populations, addressing a critical gap in the literature.

## Methods

### Study design and population

This study employed a cross-sectional design utilizing Phase 8 (2018–2020) data from the Pregnancy Risk Assessment Monitoring System (PRAMS). PRAMS is a multi-state, population-based surveillance system collaboratively managed by the Centers for Disease Control and Prevention (CDC), Division of Reproductive Health, and various state, territorial, and local health departments. The PRAMS sample comprises individuals who have recently experienced a live birth and provides comprehensive data on maternal and infant healthcare utilization and health outcomes. Data collection for PRAMS is conducted through a mailed questionnaire, followed by additional telephone follow-ups over a period of two to four weeks. All data collection and management procedures are conducted using the PRAMS Integrated Data Collection System (PIDS), developed by the CDC and securely stored on CDC servers (CDC, 2024; Shulman et al. 2018).

**Fig. 1:**
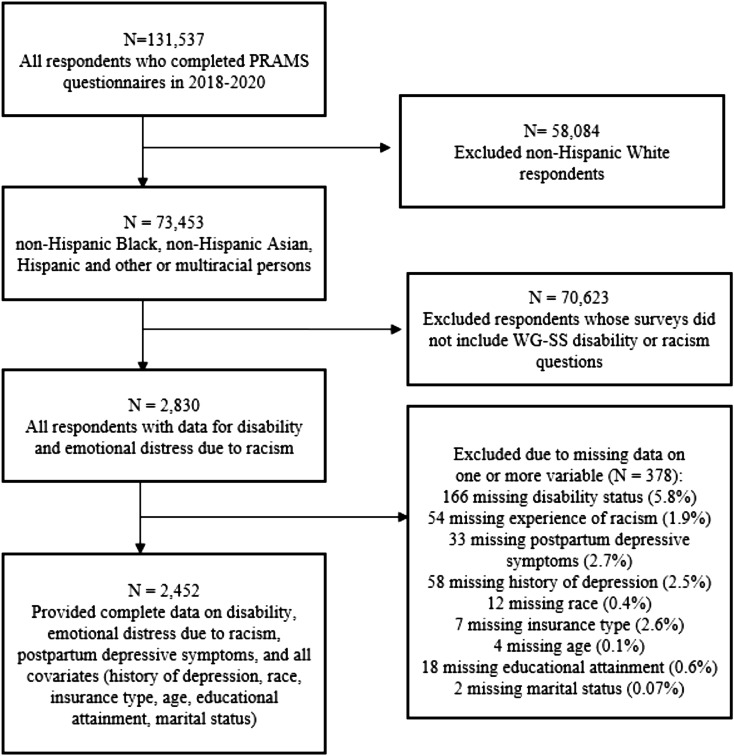
Respondent inclusion flow chart

This study focused on PRAMS respondents from states that included optional questions on both experiences of racism and disability status (i.e., MO, GA, VA) (Fig. 1). Eligible respondents were those who self-identified as members of one of the following racial or ethnic groups recorded in PRAMS: Black, Asian, American Indian, Chinese, Japanese, Filipino, Hawaiian, Alaska Native, Hispanic, other or multiracial. Because this analysis specifically examines the effects of experiences of racism for respondents who identify with a marginalized racial or ethnic group, white respondents were excluded. Racial and ethnic categories were consolidated into four broad groups: non-Hispanic Black, non-Hispanic Asian, Hispanic, and other or multiracial persons due to small sample sizes. Each state participating in PRAMS employs its own sampling methodology; thus, weighted samples are typically used to adjust for these sampling variations (CDC, 2024). However, because our study sample included respondents from three states, the sample generated was a non-representative, nonrandom subset; thus, survey weights were not applied (Bollen et al. 2016).

### Measures

The primary exposure of interest was emotional distress due to racism (EDR), assessed with the question: “During the 12 months before your new baby was born, did you feel emotionally upset (for example, angry, sad or frustrated) as a result of how you were treated based on your race?” (yes, no). The association between EDR and PPD symptoms was assessed by disability status to compare this relationship among persons with and without disabilities. In 2018, PRAMS incorporated a disability supplement consisting of the Washington Group Short Set on Functioning (WG-SS), (D’Angelo et al. 2020; Release 2018). This set of questions, based on the World Health Organization’s International Classification of Functioning, Disability and Health, provides standardized measures for disability (Madans et al. 2011; Svestková 2008). Respondents were asked if they had difficulties in the following areas of functioning: seeing (even when wearing glasses or contact lenses), hearing (even with a hearing aid), walking or climbing steps, remembering or concentrating, self-care (e.g., washing or dressing), and communicating, understanding, or being understood in their usual language. Response options included “no difficulty,” “some difficulty,” “a lot of difficulty”, and “I cannot do this at all.” Disability status was dichotomized into two levels of functional impairment: respondents who reported “no difficulty” or “some difficulty” in all assessed functional areas were classified as having “no disability”, while those who reported “a lot of difficulty” or “I cannot do this at all” in at least one functional area were classified as having “at least one disability”. This two-way disaggregation method enables the comparative analysis of outcomes between two heterogenous groups (respondents with and without disabilities) and has been recommended for assessing potential outcome variations by disability status (Hanass-Hancock et al. 2023).

### Statistical analysis

The primary outcome of interest was PPD symptoms, assessed using two key questions in PRAMS: “Since your new baby was born, how often have you felt down, depressed or hopeless?” and “Since your new baby was born, how often have you had little interest or little pleasure in doing things you usually enjoyed?” Response options included “always,” “often,” “sometimes,” “rarely,” and “never.” As aligned with methodology in prior CDC reports, participants who responded “always” or “often” to either survey question were classified as experiencing PPD symptoms while those who answered “sometimes”, “rarely,” or “never” to both questions were classified as not experiencing PPD symptoms (Ko et al. 2017).

Covariates in the analysis included maternal age (< 20, 20–24, 25–34, 35+), race and ethnicity (non-Hispanic Black, non-Hispanic Asian, Hispanic, other or multiracial), educational attainment (< high school, high school, some college, Bachelors+), marital status (married, other), insurance at delivery (Medicaid, private insurance, other), and history of depression based on previous literature (Alhusen et al. 2023; Guintivano, Manuck, et al., 2018; Weeks et al., 2022). Depression prior to pregnancy was identified if symptoms were present within three months before pregnancy, and prenatal depression was identified if respondents reported having depressive symptoms during their most recent pregnancy. Given the high correlation between pre-pregnancy and prenatal depressive symptoms, a composite variable was created to represent a history of depression, which was included in the multivariate models for PPD symptoms. History of depression was considered present if respondents reported depressive symptoms either before pregnancy or during pregnancy, and absent if respondents reported no depressive symptoms at either time point.

All statistical analyses were performed using SAS 9.4 software (Cary, NC). Surveys with incomplete data on experience of racism, disability status, history of depression, or relevant covariates were deemed missing (see Fig. 1). Since less than 5% of data were missing for all covariates, a complete case analysis was employed, aligning with prior methodological literature (Bennet, 2001; Schafer 1999). Associations between EDR and PPD symptom prevalence by disability status were evaluated using Pearson’s chi-square test. To further compare associations between EDR and PPD symptoms among persons with and without disabilities, multivariate logistic regression models were constructed. An interaction term comprising EDR and disability status was included in each model to examine variations in the relationship between EDR and PPD symptoms based on disability status, and to test the hypothesis that respondents reporting at least one disability exhibit greater odds of PPD symptoms compared to those without disabilities. Model 1 examined the unadjusted association between EDR and PPD symptoms and its interaction among respondents with and without disabilities. Model 2 adjusted for maternal demographic characteristics, including maternal age, race/ethnicity, educational attainment, marital status, and insurance payer. Finally, model 3 included adjustments for history of depression in addition to the covariates included in Model 2.

## Results

Our final sample was comprised of 2,452 non-Hispanic Black (*n* = 1455), non-Hispanic Asian (*n* = 216), Hispanic (*n* = 586), and other or multiracial (*n* = 195) respondents; of which 162 (6.6%) respondents reported at least one disability. Full demographic characteristics are summarized in Table 1. A total of 311 (12.7%) respondents experienced EDR in the year prior to pregnancy including 44 (14.1%) respondents with disabilities. Those who experienced EDR were more likely to be non-Hispanic Black (60.1%), aged 25 to 34 years (59.2%), and not married (59.8%). Approximately half of respondents (49.8%) who experienced EDR also reported having Medicaid as a primary insurance payer. Overall, those who experienced EDR demonstrated a higher rate of PPD symptoms (24.4%) compared to those who did not experience EDR (13.8%).

**Table 1:** Respondent characteristics by emotional distress due to racism within one year prior to delivery among persons in Racial and ethnic minoritized populations who delivered a live infant in three PRAMS participating states, 2018–2020

Characteristics	Total: *N* = 2452	No Emotional Distress Due to Racism (*N* = 2141)	Emotional Distress Due to Racism (*N* = 311)
	n (Column %)	n (Column %)	n (Column %)
Disability Status			
No disability	2290(93.4%)	2023(94.5%)	267(85.9%)
At least one disability	162(6.6%)	118(5.5%)	44(14.1%)
Maternal Age			
< 20 years	530(21.6%)	484(22.6%)	46(14.8%)
20–24 years	130(5.3%)	113(5.3%)	17(5.5%)
25–34 years	1344(54.8%)	1160(54.2%)	184(59.2%)
35 + years	448(18.3%)	384(17.9%)	64(20.6%)
Maternal Race			
Asian, non- HS	216(8.8%)	198(9.2%)	18(5.8%)
Black, non-HS	1455(59.3%)	1268(59.2%)	187(60.1%)
Hispanic	586(23.9%)	508(23.7%)	78(25.1%)
Other/Multiracial	195(8%)	167(7.8%)	28(9%)
Educational Attainment			
< High School	320(13.1%)	286(13.4%)	34(10.9%)
High School	879(35.8%)	777(36.3%)	102(32.8%)
Some College	687(28%)	604(28.2%)	83(26.7%)
Bachelor’s +	566(23.1%)	474(22.1%)	92(29.6%)
Marital Status			
Married	980(40%)	855(39.9%)	125(40.2%)
Other	1472(60%)	1286(60.1%)	186(59.8%)
Insurance Type			
Medicaid	1352(55.1%)	1197(55.9%)	155(49.8%)
Private	845(34.5%)	723(33.8%)	122(39.2%)
Other	255(10.4%)	221(10.3%)	34(10.9%)
History of Depression			
No	1989(81.1%)	1802(84.2%)	187(60.1%)
Yes	463(18.9%)	339(15.8%)	124(39.9%)
Postpartum Depressive Symptoms			
No	2080(84.8%)	1845(86.2%)	235(75.6%)
Yes	372(15.2%)	296(13.8%)	76(24.4%)

Table 2 displays the results of the chi-square test of association between EDR and PPD symptom prevalence among those with and without disabilities. This unadjusted analysis revealed a significant association between EDR and PPD symptoms in both groups: respondents with disabilities (*p* = 0.01) and those without disabilities (*p* = 0.002). Specifically, respondents with EDR demonstrated a higher prevalence of PPD symptoms compared to those without EDR in both groups. Importantly, the prevalence of PPD symptoms associated with EDR was notably higher among respondents with disabilities (52.3%) compared to respondents without disabilities (19.9%), demonstrating a potential effect of disability status and EDR on PPD outcomes.

**Table 2:** Associations between emotional distress due to racism and postpartum depression symptoms among persons with and without disabilities in Racial and ethnic minoritized populations who delivered a live infant in three PRAMS participating states, 2018–2020

			No Postpartum Depression Symptoms	Postpartum Depression Symptoms	Chi-Square *p*-value
Disability status			n (Row %)	n (Row %)	
No disability	No emotional distress due to racism	2023	1764(87.2)	259 (12.8)	0.0016
	Emotional distress due to racism	267	214 (80.2)	53 (19.9)	
	Total	2290			
At least one disability	No emotional distress due to racism	118	81 (68.6)	37 (31.4)	0.01
	Emotional distress due to racism	44	21 (47.7)	23 (52.3)	
	Total	162			

Table 3 presents the association between experiences of EDR and PPD symptoms, adjusted for maternal characteristics and history of depression. Notably, both EDR and the presence of at least one disability were identified as independent predictors of PPD symptoms prior to subsequent regression analyses (see Supplemental Table 1). In the unadjusted model (Model 1), respondents with disabilities who reported experiencing EDR had more than twice the odds of exhibiting PPD symptoms compared to respondents with disabilities who did not report EDR (OR: 2.398, 95% CI: 1.181–4.867). Among respondents without disabilities, EDR was associated with a 70% increase in the odds of PPD symptoms compared to those without disabilities who did not experience EDR (OR: 1.687, 95% CI: 1.215–2.341). In model 2, which adjusted for maternal demographic factors (i.e., age, race/ethnicity, educational attainment, marital status, and insurance payer), respondents with disabilities who reported EDR had 2.5 times the odds of experiencing PPD symptoms compared to those with disabilities who did not report EDR (aOR: 2.490, 95% CI: 1.211–5.120). Among those without disabilities, EDR was associated with a 90% increase in the odds of PPD symptoms compared to those without disabilities who did not experience EDR (aOR: 1.890, 95% CI: 1.351–2.643). Across Models 1 and 2, EDR remained significantly associated with increased odds of PPD symptoms in both the disability and no disability group. However, an interaction between disability status and EDR did not achieve statistical significance, indicating that the association between EDR and PPD symptoms did not differ significantly by disability group (see Supplemental Table 1). In the fully adjusted model (Model 3), which controlled for both maternal characteristics and history of depression, the association between EDR and PPD symptoms, as analyzed by disability status, was no longer statistically significant. This attenuation suggests a significant effect of prior depressive symptoms on PPD risk across respondents with and without disabilities.

**Table 3:** Adjusted associations of emotional distress due to racism and postpartum depression symptoms among persons with and without disabilities in minoritized Racial and ethnic populations who delivered a live infant in three PRAMS participating states, 2018–2020

	Comparison	Odds Ratio	95% Confidence Interval		*p*-value for interaction
Model 1*	EDR among respondents with at least one disability (ref = no EDR among respondents with at least one disability)	2.398	1.181	4.867	0.377
	EDR among respondents with no disability (ref = no EDR among respondents with no disability)	1.687	1.215	2.341	---
Model 2**	EDR among respondents with at least one disability (ref = no EDR among respondents with at least one disability)	2.490	1.211	5.120	0.496
	EDR among respondents with no disabilities (ref = no EDR among respondents with no disability)	1.890	1.351	2.643	---
Model 3***	EDR among respondents with at least one disability (ref = no EDR among respondents with at least one disability)	1.806	0.855	3.814	0.565
	EDR among respondents with no disability (ref = no EDR among respondents with no disability)	1.418	0.998	2.017	---

*Model included EDR, disability status, and their interaction

**Model adjusted for demographics: age, race/ethnicity, educational attainment, marital status, and insurance payer

*** Model adjusted for demographics (as above) and history of depression

## Discussion

This population-based study is the first to investigate the association between experiences of EDR and PPD symptoms among persons with and without disabilities in minoritized racial and ethnic populations. The findings contribute to the existing body of research demonstrating that EDR in minoritized racial and ethnic groups is associated with an increased risk of PPD symptoms, with a higher prevalence of PPD symptoms observed among those with disabilities compared to those without disabilities (Bossick et al. 2022; Weeks et al. 2022). While prior research has established a link between EDR exposure and heightened PPD risk, this is the first study to examine variations in this association based on the intersection of minoritized race/ethnic identity and disability status (Bossick et al. 2022; Bower et al. 2023; Weeks et al., 2022).

In this study, the prevalence of EDR across all respondents closely aligned with previously reported estimates derived from PRAMS data (Bossick et al. 2022; Bower et al. 2023; Weeks et al., 2022). In a large population-based study of PRAMS respondents from 11 states and New York City, Bossick et al. (2022) found that 11.8% of postpartum people of color experienced EDR. Similarly, in this study, we found that an estimated 12.7% of respondents from minoritized racial and ethnic groups across three PRAMS-participating states (MO, GA, VA) reported experiences of EDR in the year prior to pregnancy.

The prevalence of EDR in this study was notably higher among respondents identifying as non-Hispanic Black, those who were unmarried, and those utilizing Medicaid as their primary health insurance payer. These results are consistent with prior research demonstrating that self-reported experiences of racial discrimination vary by racial and ethnic identity, as well as other maternal demographic characteristics (Magaña et al. 2016; Mohamoud et al., 2023). An analysis of discrimination in maternity care found that self-reported racial discrimination was particularly prevalent among Black (12.9%) and multiracial (10.6%) individuals compared to White (1.6%), Hispanic (7.3%), Asian (6.1%), and American Indian, Alaska Native, Pacific Islander, or Native Hawaiian (8.6%) individuals (Mohamoud et al., 2023). Furthermore, race-related discrimination experiences were more frequently reported among those without health insurance (28.1%) or public insurance (26.1%) compared to those with private insurance (15.9%) (Mohamoud et al., 2023).

Regarding persons with disabilities, Magaña et al. (2016) found that non-Latino Black and Latino persons with intellectual and other developmental (IDD) disabilities were more likely to have lower educational attainment, reside in urban areas, and lack adequate health insurance coverage compared to their non-Latino White counterparts with IDD and non-Latino Black and Latino persons without IDD. These socioeconomic disparities, influenced by both racial/ethnic identity and disability status, were linked to worse mental and physical health outcomes among non-Latino Black and Latino persons with IDD (Magaña et al. 2016). These findings, and other corroborating studies, have emphasized the need for additional research employing an intersectional perspective to better understand the compounded influences of racial and disability-based disparities in health outcomes (Nuru-Jeter et al. 2011; Varadaraj et al. 2021).

In the present study, approximately one in seven respondents from minoritized racial and ethnic populations with disabilities reported experiencing EDR. Prior to examining associations between EDR and PPD symptoms across disability groups, we found that both EDR and disability status independently predicted PPD symptoms, consistent with previous research (Alhusen et al. 2023; Bossick et al. 2022; Bower et al. 2023; Mitra et al. 2015). Additionally, our unadjusted analyses revealed an association between EDR and PPD symptoms, with a higher estimated prevalence of PPD symptoms among respondents with disabilities who experienced EDR (52.3%) compared to those without disabilities who experienced EDR (19.9%). These findings suggest that respondents with disabilities who experienced EDR may be particularly vulnerable to PPD symptoms compared to those without disabilities who experienced EDR. Contrary to our hypothesis, however, we did not find a significant interaction between EDR and disability status in our unadjusted and adjusted models. EDR emerged as a significant predictor of PPD symptoms across both disability groups, with no significant difference in the strength of the association between EDR and PPD symptoms when comparing persons with and without disabilities.

Notably, history of depression influenced the association between EDR and PPD outcomes across both disability groups, suggesting that respondents with prior depressive episodes who experienced EDR may be at an elevated risk for PPD symptoms. This finding may be particularly salient for persons with disabilities within minoritized racial and ethnic populations, as prior research indicates that both disability status and minoritized racial/ethnic identity are associated with an increased likelihood of experiencing discrimination and history of depression (Artiga et al. 2023; Bower et al. 2023; Chen et al. 2023). Importantly, persons with disabilities from minoritized racial and ethnic backgrounds are disproportionately exposed to social stressors, including stigma and socioeconomic disadvantage, which may further contribute to an increased vulnerability to PPD and depression across the lifespan (Altman et al., 2008).

### Future research directions

The findings in this study emphasize the need for further research investigating disparities in PPD outcomes, particularly among persons with disabilities in minoritized racial and ethnic groups. Prior evidence indicates a higher prevalence of disability among persons in minoritized racial and ethnic groups, suggesting a population at increased risk for health-related disparities (Goyat et al., 2016; Varadaraj et al. 2021). Furthermore, persons at the intersection of disability and minoritized racial/ethnic identity are disproportionately impacted by adverse social determinants of health, including systemic discrimination, which can contribute to adverse maternal health outcomes, including PPD (Dorsey Holliman et al. 2023; Mereish 2012).

Further research utilizing larger, population-based samples is needed to elucidate potential disparities in the relationship between experiences of discrimination and PPD symptoms among persons with disabilities in minoritized racial and ethnic populations. While this study identified a higher prevalence of PPD symptoms among persons with disabilities who experienced EDR, a significant interaction between disability status and EDR was not observed. A lack of observed interaction may be attributable to small cell sizes and lack of precision and should be corroborated by larger population-based studies. Additionally, the aggregation of variables due to small cell sizes, including disability type, severity of the disability, and racial/ethnic categorization, may obscure important nuances in the relationship between EDR and PPD outcomes and warrants additional research using disaggregated measures in larger sample sizes (Hanass-Hancock et al. 2023).

In addition to larger population-based studies, additional research is needed to examine the impact of exposure to various forms of discrimination for those with multiple marginalized social identities. While the present study examined disparities in PPD outcomes related to experiences of racism, we recommend that future studies also assess the impact of ableism which was not addressed by PRAMS survey questions during the present study. Additionally, the influence of discrimination based on gender, sexual orientation, and socioeconomic status warrants further investigation, as these factors may significantly contribute to health disparities among persons with disabilities (Varadaraj et al. 2021). As a result of continued advocacy from our team and others, PRAMS methodology now requires the WG-SS in all states, and we continue to encourage broader data collection for better understanding of health disparities among persons with disabilities across all racial and ethnic populations and in diverse subpopulations.

### Strengths and limitations

There are limitations of this study that must be acknowledged. First, since PRAMS relies on retrospective, self-reported survey data, the results may be affected by item misunderstanding, recall bias, social desirability bias, and other potential biases. Second, the questions regarding both racism and disability status were asked only in three states, resulting in a relatively small sample size that limits the generalizability of our findings. Small cell sizes and/or inadequate statistical power can hinder the detection of potential interactions and, even with a large overall sample size, obtaining stable estimates for rare categorical outcomes in smaller subgroups can be difficult after covariate adjustment. We, again, advocate for broader data collection to better elucidate disparities among persons in racial and ethnic subpopulations. Finally, certain disabilities may be underreported due to the language used in the PRAMS questionnaire. For example, individuals on the autism spectrum may require greater specificity or have difficulty responding to Likert-type questions, and those with intellectual disabilities may need simplified vocabulary or shorter sentences (Nicolaidis et al. 2015). This consideration is crucial given the growing evidence that people with intellectual and developmental disabilities are at heightened risk of adverse perinatal outcomes (Mitra et al. 2021).

Nonetheless, this study possesses notable strengths. This is the first known study to employ an intersectional framework to analyze PPD outcomes among persons with and without disabilities in minoritized racial and ethnic populations, a demographic that has been historically excluded from research. Additionally, this study utilized data from PRAMS, one of the largest and most comprehensive databases available for investigating research questions related to perinatal outcomes. Using multivariable regression analyses, we investigated the relationship between EDR and PPD symptoms, considering the potential interaction effects of both EDR and disability status on PPD outcomes. Methodologically, examining the joint effects of multiple individual variables (e.g., experience of EDR and disability status) is widely utilized in intersectionality studies and recommended by scholars (Guan et al. 2021). Furthermore, this study examined outcomes related to experiences of discrimination, a core component of intersectionality, with implications for greater advocacy and social equity for underserved populations (Bowleg 2012; Hoang and Wong 2022).

## Conclusion

Postpartum depression is a prevalent adverse outcome which disproportionately affects persons in minoritized racial and ethnic populations and persons with disabilities. Our findings provide an important step in understanding additional disparities in PPD outcomes among persons with disabilities in minoritized racial and ethnic groups. Large population-based studies employing an intersectional lens are needed to elucidate disparities in PPD outcomes related to multiple, overlapping social identities and experiences of discrimination.

## References

[CR1] Alhusen JL, Hughes RB, Lyons G, Laughon K (2023) Depressive symptoms during the perinatal period by disability status: findings from the united States pregnancy risk assessment monitoring system. J Adv Nurs 79(1):223–233. 10.1111/jan.15482 36320150 10.1111/jan.15482 PMC9795828

[CR2] Allen AM, Prince CB, Dietz PM (2009) Postpartum depressive symptoms and smoking relapse. Am J Prev Med 36(1):9–12. 10.1016/j.amepre.2008.09.020 19095161 10.1016/j.amepre.2008.09.020

[CR3] Altman B, Bernstein A (2008) Disability and health in the united states, 2001–2005. National Center for Health Statistics, Hyattsville, MD

[CR4] American Psychiatric Association (2013) Diagnostic and statistical manual of mental disorders: DSM-5™, 5th ed (pp. xliv, 947). American Psychiatric Publishing, Inc. 10.1176/appi.books.9780890425596

[CR5] Artiga S, Hamel L, Gonzalez-Barrera A, Montero A, Hill L, Presiado M, Kirzinger A, Published LL (2023), December 5 Survey on Racism, Discrimination and Health - Findings– 10257. KFF. https://www.kff.org/report-section/survey-on-racism-discrimination-and-health-findings/

[CR6] Bauman BL (2020) Vital Signs: Postpartum Depressive Symptoms and Provider Discussions About Perinatal Depression — United States, 2018. MMWR. Morbidity and Mortality Weekly Report, 69. 10.15585/mmwr.mm6919a2 10.15585/mmwr.mm6919a2 PMC7238954 32407302

[CR7] Bennett DA (2001) How can I deal with missing data in my study? Aust N Z J Public Health 25(5):464–469. 10.1111/j.1467-842X.2001.tb00294.x 11688629

[CR8] Bollen KA, Biemer PP, Karr AF, Tueller S, Berzofsky ME (2016) Are survey weights needed?? A review of diagnostic tests in regression analysis. Annual Rev Stat its Application 3(1):375–392. 10.1146/annurev-statistics-011516-012958

[CR9] Bossick AS, Bossick NR, Callegari LS, Carey CM, Johnson H, Katon JG (2022) Experiences of racism and postpartum depression symptoms, care-seeking, and diagnosis. Archives Women’s Mental Health 25(4):717–727. 10.1007/s00737-022-01232-w 10.1007/s00737-022-01232-w 35504987

[CR10] Bower KM, Geller RJ, Jeffers N, McDonald M, Alhusen J (2023) Experiences of racism and perinatal depression: findings from the pregnancy risk assessment monitoring system, 2018. J Adv Nurs 79(5):1982–1993. 10.1111/jan.15519 36630188 10.1111/jan.15519

[CR11] Bowleg L (2012) The problem with the phrase *Women and Minorities*: Intersectionality—An important theoretical framework for public health. Am J Public Health 102(7):1267–1273. 10.2105/AJPH.2012.300750 22594719 10.2105/AJPH.2012.300750 PMC3477987

[CR12] CDC. (2024), May 20 *Data Methodology*. Pregnancy Risk Assessment Monitoring System (PRAMS). https://www.cdc.gov/prams/php/methodology/index.html

[CR13] Chen X, Lu E, Stone SL, Thu Bui OT, Warsett K, Diop H (2023) Stressful life events, postpartum depressive symptoms, and partner and social support among pregnant people with disabilities. Women’s Health Issues 33(2):167–174. 10.1016/j.whi.2022.10.006 36463011 10.1016/j.whi.2022.10.006

[CR14] Courtney-Long EA, Romano SD, Carroll DD, Fox MH (2017) Socioeconomic factors at the intersection of race and ethnicity influencing health risks for people with disabilities. J Racial Ethnic Health Disparities 4(2):213–222. 10.1007/s40615-016-0220-5 10.1007/s40615-016-0220-5 PMC5055843 27059052

[CR15] Crenshaw K (1991) Mapping the margins: intersectionality, identity politics, and violence against women of color. Stanford Law Rev 43(6):1241. 10.2307/1229039

[CR16] D’Angelo DV, Cernich A, Harrison L, Kortsmit K, Thierry JM, Folger S, Warner L (2020) Disability and pregnancy: A Cross-Federal agency collaboration to collect Population-Based data about experiences around the time of pregnancy. J Women’s Health 29(3):291–296. 10.1089/jwh.2020.8309 10.1089/jwh.2020.8309 PMC7097685 32186964

[CR17] Darcy JM, Grzywacz JG, Stephens RL, Leng I, Clinch CR, Arcury TA (2011) Maternal depressive symptomatology: 16-Month Follow-up of infant and maternal Health-Related quality of life. J Am Board Family Med 24(3):249–257. 10.3122/jabfm.2011.03.100201 10.3122/jabfm.2011.03.100201 PMC3114440 21551396

[CR18] Deierlein AL, Antoniak K, Chan M, Sassano C, Stein CR (2021) Pregnancy-related outcomes among women with physical disabilities: A systematic review. Paediatr Perinat Epidemiol 35(6):758–778. 10.1111/ppe.12781 34431112 10.1111/ppe.12781

[CR19] Dorsey Holliman B, Stransky M, Dieujuste N, Morris M (2023) Disability doesn’t discriminate: health inequities at the intersection of race and disability. Front Rehabilitation Sci 4:1075775 10.3389/fresc.2023.1075775 PMC10357509 37484601

[CR20] Feldman R, Granat A, Pariente C, Kanety H, Kuint J, Gilboa-Schechtman E (2009) Maternal depression and anxiety across the postpartum year and infant social engagement, fear regulation, and stress reactivity. J Am Acad Child Adolesc Psychiatry 48(9):919–927. 10.1097/CHI.0b013e3181b21651 19625979 10.1097/CHI.0b013e3181b21651

[CR21] Getahun D, Oyelese Y, Peltier M, Yeh M, Chiu VY, Takhar H, Khadka N, Mensah N, Avila C, Fassett MJ (2023) Trends in postpartum depression by race/ethnicity and Pre-pregnancy body mass index. Am J Obstet Gynecol 228(1):S122–S123. 10.1016/j.ajog.2022.11.248

[CR22] Goyat R, Vyas A, Sambamoorthi U (2016) Racial/Ethnic disparities in disability prevalence. J Racial Ethnic Health Disparities 3(4):635–645. 10.1007/s40615-015-0182-z 10.1007/s40615-015-0182-z PMC4919210 27294757

[CR23] Guan A, Thomas M, Vittinghoff E, Bowleg L, Mangurian C, Wesson P (2021) An investigation of quantitative methods for assessing intersectionality in health research: A systematic review. SSM - Popul Health 16:100977. 10.1016/j.ssmph.2021.100977 34869821 10.1016/j.ssmph.2021.100977 PMC8626832

[CR24] Guintivano J, Manuck T, Meltzer-Brody S (2018) Predictors of postpartum depression: A comprehensive review of the last decade of evidence. Clin Obstet Gynecol 61(3):591–603. 10.1097/GRF.0000000000000368 29596076 10.1097/GRF.0000000000000368 PMC6059965

[CR25] Hanass-Hancock J, Kamalakannan S, Murthy GVS, Palmer M, Pinilla-Roncancio M, Rivas Velarde M, Tetali S, Mitra S (2023) What cut-off(s) to use with the Washington group short set of questions? Disabil Health J 16(4):101499. 10.1016/j.dhjo.2023.101499 37481353 10.1016/j.dhjo.2023.101499

[CR26] Hoang T-MH, Wong A (2022) Exploring the application of intersectionality as a path toward equity in perinatal health: A scoping review. Int J Environ Res Public Health 20(1):685. 10.3390/ijerph20010685 36613005 10.3390/ijerph20010685 PMC9819722

[CR27] Horner-Johnson W (2020) Disability, intersectionality, and inequity: life at the margins. In Public health perspectives on disability. Springer US 91–105. 10.1007/978-1-0716-0888-3_4

[CR28] Ko JY, Rockhill KM, Tong VT, Morrow B, Farr SL (2017) Trends in Postpartum Depressive Symptoms—27 States, 2004, 2008, and 2012. MMWR. Morbidity and Mortality Weekly Report, 66. 10.15585/mmwr.mm6606a1 10.15585/mmwr.mm6606a1 PMC5657855 28207685

[CR29] Lilja G, Edhborg M, Nissen E (2012) Depressive mood in women at childbirth predicts their mood and relationship with infant and partner during the first year postpartum: depressive mood in women at childbirth. Scand J Caring Sci 26(2):245–253. 10.1111/j.1471-6712.2011.00925.x 21950600 10.1111/j.1471-6712.2011.00925.x

[CR30] Lubotzky-Gete S, Ornoy A, Grotto I, Calderon-Margalit R (2021) Postpartum depression and infant development up to 24 months: A nationwide population-based study. J Affect Disord 285:136–143. 10.1016/j.jad.2021.02.042 33647581 10.1016/j.jad.2021.02.042

[CR31] Madans JH, Loeb ME, Altman BM (2011) Measuring disability and monitoring the UN convention on the rights of persons with disabilities: the work of the Washington group on disability statistics. BMC Public Health 11(Suppl 4):S4. 10.1186/1471-2458-11-S4-S4 21624190 10.1186/1471-2458-11-S4-S4 PMC3104217

[CR32] Magaña S, Parish S, Morales MA, Li H, Fujiura G (2016) Racial and ethnic health disparities among people with intellectual and developmental disabilities. Intellect Dev Disabil 54(3):161–172. 10.1352/1934-9556-54.3.161 27268472 10.1352/1934-9556-54.3.161

[CR33] Mereish EH (2012) The intersectional invisibility of race and disability status: an exploratory study of health and discrimination facing Asian Americans with disabilities. Ethn Inequalities Health Social Care 5(2):52–60

[CR35] Mitra M, Iezzoni LI, Zhang J, Long-Bellil LM, Smeltzer SC, Barton BA (2015) Prevalence and risk factors for postpartum depression symptoms among women with disabilities. Matern Child Health J 19(2):362–372. 10.1007/s10995-014-1518-8 24889114 10.1007/s10995-014-1518-8 PMC4254905

[CR34] Mitra M, Akobirshoev I, Valentine A, Brown HK, Moore Simas TA (2021) Severe maternal morbidity and maternal mortality in women with intellectual and developmental disabilities. Am J Prev Med 61(6):872–881. 10.1016/j.amepre.2021.05.041 34579985 10.1016/j.amepre.2021.05.041 PMC8608722

[CR36] Mohamoud YA (2023) Vital Signs: Maternity Care Experiences — United States, April 2023. MMWR. Morbidity and Mortality Weekly Report, 72. 10.15585/mmwr.mm7235e1 10.15585/mmwr.mm7235e1 37651304

[CR37] Nicolaidis C, Raymaker D, Katz M, Oschwald M, Goe R, Leotti S, Grantham L, Plourde E, Salomon J, Hughes RB, Powers LE (2015) Community-Based participatory research to adapt health measures for use by people with developmental disabilities. Progress Community Health Partnerships: Res Educ Action 9(2):157–170. 10.1353/cpr.2015.0037 10.1353/cpr.2015.0037 26412758

[CR38] Nuru-Jeter AM, Thorpe RJ, Fuller-Thomson E (2011) Black-White differences in Self-Reported disability outcomes in the U.S.: early childhood to older adulthood. Public Health Rep 126(6):834–843. 10.1177/003335491112600609 22043099 10.1177/003335491112600609 PMC3185319

[CR39] Onyewuenyi TL, Peterman K, Zaritsky E, Ritterman Weintraub ML, Pettway BL, Quesenberry CP, Nance N, Surmava A-M, Avalos LA (2023) Neighborhood disadvantage, race and ethnicity, and postpartum depression. JAMA Netw Open 6(11):e2342398. 10.1001/jamanetworkopen.2023.42398 37955900 10.1001/jamanetworkopen.2023.42398 PMC10644210

[CR40] Paris R, Bolton RE, Weinberg MK (2009) Postpartum depression, suicidality, and mother-infant interactions. Archives Women’s Mental Health 12(5):309–321. 10.1007/s00737-009-0105-2 10.1007/s00737-009-0105-2 19728036

[CR41] Release (2018), November 1: NIH to fund national data collection on new mothers with disabilities| NICHD - Eunice Kennedy Shriver National Institute of Child Health and Human Development. https://www.nichd.nih.gov/newsroom/news/110118-PRAMS

[CR42] Schafer JL (1999) Multiple imputation: a primer. Stat Methods Med Res 8(1):3–15. 10.1177/096228029900800102 10347857 10.1177/096228029900800102

[CR43] Shulman HB, D’Angelo DV, Harrison L, Smith RA, Warner L (2018) The pregnancy risk assessment monitoring system (PRAMS): overview of design and methodology. Am J Public Health 108(10):1305–1313. 10.2105/AJPH.2018.304563 30138070 10.2105/AJPH.2018.304563 PMC6137777

[CR44] Slomian J, Honvo G, Emonts P, Reginster J-Y, Bruyère O (2019) Consequences of maternal postpartum depression: A systematic review of maternal and infant outcomes. Women’s Health 15:174550651984404. 10.1177/1745506519844044 10.1177/1745506519844044 PMC6492376 31035856

[CR45] Svestková O (2008) International classification of functioning, disability and health of world health organization (ICF). Prague Med Rep 109(4):268–274 19537677

[CR46] Tarasoff LA (2017) We don’t know. We’ve never had anybody like you before: barriers to perinatal care for women with physical disabilities. Disabil Health J 10(3):426–433. 10.1016/j.dhjo.2017.03.017 28404229 10.1016/j.dhjo.2017.03.017

[CR47] Varadaraj V, Deal JA, Campanile J, Reed NS, Swenor BK (2021) National prevalence of disability and disability types among adults in the US, 2019. JAMA Netw Open 4(10):e2130358. 10.1001/jamanetworkopen.2021.30358 34673966 10.1001/jamanetworkopen.2021.30358 PMC8531993

[CR48] Weeks F, Zapata J, Rohan A, Green T (2022) Are experiences of Racial discrimination associated with postpartum depressive symptoms?? A multistate analysis of pregnancy risk assessment monitoring system data. J Women’s Health 31(2):158–166. 10.1089/jwh.2021.0426 10.1089/jwh.2021.0426 PMC10941332 34967671

